# Mental health service contact following release from prison or hospital discharge in those with psychosis

**DOI:** 10.3389/fpsyt.2022.1034917

**Published:** 2022-12-15

**Authors:** Nabila Z. Chowdhury, Handan Wand, Olayan Albalawi, Armita Adily, Azar Kariminia, Stephen Allnutt, Grant Sara, Kimberlie Dean, Andrew Ellis, David Greenberg, Peter W. Schofield, Tony Butler

**Affiliations:** ^1^School of Population Health, University of New South Wales, Sydney, NSW, Australia; ^2^The Kirby Institute, University of New South Wales, Sydney, NSW, Australia; ^3^Department of Statistics, Faculty of Science, University of Tabuk, Tabuk, Saudi Arabia; ^4^Forensic Mental Health Program, University of New South Wales, Sydney, NSW, Australia; ^5^Faculty of Medicine and Health, University of Sydney, NSW, Australia; ^6^Discipline of Psychiatry and Mental Health, School of Clinical Medicine, Faculty of Medicine and Health, University of New South Wales and Justice Health and Forensic Mental Health Network, Sydney, NSW, Australia; ^7^NSW Justice Health & Forensic Mental Health Network, Newcastle, NSW, Australia; ^8^The University of Newcastle, Newcastle, NSW, Australia

**Keywords:** post-release, prison, mental health, treatment, psychiatric care

## Abstract

**Background:**

An association exists between psychosis and criminal offending, which evidence suggests can be reduced by effective mental health care for this vulnerable population. However mental health services often lose contact with people after diagnosis. The association between the first episode of psychosis and criminal offending highlights the need for effective mental health care for this vulnerable population.

**Aims:**

To investigate the association between the first diagnosis of psychosis (FDP) in prison or hospital and subsequent mental health service contact following release from prison or discharge from hospital.

**Materials and methods:**

Individuals with a FDP either in prison (*n* = 492) or hospital setting (*n* = 24,910) between July 2006 and December 2011 in NSW (Australia), were followed post-release or discharge until their first mental health service contact in the community, the occurrence of an offence, death, or completion of the study period at the end of December 2012. Cox regression models were used to examine the predictors for the mental health service contacts following release or discharge.

**Results:**

Over 70% of those with a FDP in prison or hospital had a psychosis-related or any *community-based* mental health service contact following release or discharge between July 2006 and December 2012. Those with a FDP in prison were more likely to have no contact with mental health services than those in hospital with no prior offence record (hazard ratio, *HR* = 3.14, 95% CI: 2.66–3.72 and adjusted hazard ratio, *aHR* = 3.05, 95% CI: 2.56–3.63) within a median follow-up time of 25 days for the prison group and 26 days for hospital group. Males, individuals of Aboriginal heritage and individuals diagnosed with substance-related psychoses compared to those with schizophrenia and related psychoses were less likely to have a mental health service contact following release or discharge in both the univariable and multivariable analysis.

**Conclusion:**

This study suggests that prior offending or a previous prison episode represents a barrier to mental health service contact in the community for those with a FDP. Effective rehabilitation planning while exiting prison and discharge planning from hospital are essential to the successful reintegration of these individuals with a FDP.

## Introduction

Reintegration back into the community following incarceration poses many challenges with studies showing poor health, social and justice outcomes for those released from prison (1, 2). After release into the community, many face major challenges including unstable housing, stigma, reconnecting with family and friends, and financial instability, all of which can impact on health. A study of newly released prisoners from Massachusetts state prisons found over half were unemployed and many depended on family members for housing and financial support within 1 year of release (3). One Australian study of individuals released from prison reported that having stable housing post-release was associated with better physical and psychological health (4).

The immediate post-release period has also been shown to be linked to an increased risk of suicide and drug overdose, highlighting the post-release vulnerability of this population (5). These studies indicate the importance of effective pre-release planning and post-release support for prisoners to ensure a successful return to the community.

Those with mental illness are likely to find additional challenges during this period. A retrospective study of male prisoners in Canada who had at least one major mental disorder reported that engagement with community mental health services was associated with lower recidivism (6). An Australian population-based data-linkage study of 7,030 offenders (70% male) with psychosis showed that, in men, there was an association between increased contact with community mental health services within 30 days after their index offence and reduced reoffending (7). According to a study of 1,216 sentenced prisoners from Queensland, Australia (August 2008–July 2010), reduced community mental health service contacts were reported in the year following release from prison in those who were highly psychologically distressed (8). These findings support the notion that contact with mental health services in the community may be a useful strategy for reducing reoffending and returns to prison. Little is known about reintegration issue in those prisoners with a FDP occurring whilst in prison. Many studies report strong associations between the first episode of psychosis and violent crime (9, 10), and that preventing offending can be achieved by shortening the duration of untreated psychosis (DUP) (11).

Discharge from mental health facilities has similarly been identified as problematic. In a systematic review of 45 articles high rates of negative consequences in the patients’ life which included: suicide, violent behaviour, re-hospitalisation, social maladjustment, and stigma occurred following discharge back to the community (12). A systematic review of interventions for adults admitted to hospitals with mental illness identified the need for pre- and post-discharge patient psychoeducation, structured needs assessments, medication reconciliation education, transition managers, and in-patient or out-patient provider communication to ensure a successful transition back to the community and prevent relapse and re-admission to the hospital (13). Similar to the increased suicide risk found among those released from prison, a higher risk of suicide during the first few weeks has also been reported among those discharged from psychiatric care (14).

Despite the post-release and post-discharge periods both separately being identified as risk periods, previous research has not compared post-release or post-discharge access to mental health services in the community in those with severe mental illness. In this study, we compared mental health service use in the community in those with a FDP in prison and released from prison with those with a FDP in hospital and discharged into the community in New South Wales (NSW), Australia. We further analysed the characteristics of mental health service contacts of the study groups by prior offence or incarceration history.

## Materials and methods

### Study population

We selected all individuals with a FDP in prison or hospital (public or private) between July 2006 and December 2011 in NSW (Australia) and released or discharged to the community during this time period. Individuals were followed from the date of release or discharge from prison or hospital until the occurrence of their first mental health service contact in the community, i.e., either hospital admission or emergency department presentation for a psychosis-related diagnosis or contact with community mental health service centres; or the occurrence of an offence (charged with an offence but not necessarily leading to incarceration) or death or end of the study period (December 2012). Further, we divided those with a FDP in hospital into two sub-groups depending on whether there was any prior criminal offence recorded in the state’s Reoffending Database administered by the NSW Bureau of Crime Statistics and Research (15).

### Source of data

We used de-identified, whole-of-population administrative data linked across several NSW health and justice systems. We used the NSW Ministry of Health’s Admitted Patient Data Collection (APDC), Emergency Department Data Collection (EDDC), and Mental Health Ambulatory Data (MH-AMB) for all diagnostic records of psychosis and a range of demographic variables. The NSW Reoffending Database (RoD) was used to obtain information on any criminal conviction of the study population. We used data from NSW Offender Integrated Management System (OIMS) for incarceration records of sentenced prisoners. The NSW Registry of Births Deaths and Marriages (RBDM) was used to identify deaths in the study population.

#### Selection of individuals with a first diagnosis of psychosis

Individuals with a FDP were identified using an algorithm adapted from Sara and Malhi (16) for bipolar disorder where a first diagnosis of bipolar disease was defined using a maximum 5-year period during which no diagnosis was recorded (16). This approach allowed us to infer that this was likely the first treatment episode for psychosis of our study population.

Psychosis was identified according to the International Classification of diseases 9th and 10th Revision (ICD-9 and ICD-10 codes) (17, 18) and mapped to the relevant Systematized Nomenclature of Medicine–Clinical Terms (SNOMED CT) codes. Mapping was done by the National Clinical Terminology and Information Service, Australian Digital Health Agency. Psychotic disorders included schizophrenia and related psychoses (ICD-10 codes F20, F22–F25, F28, and F29; ICD-9 code 295), affective psychoses (ICD-10 codes F30.2, F31.2, F31.5, F32.3, and F33.3; ICD-9 codes 296.8 and 296.9), and substance-related psychoses (ICD-10 codes F10.5, F11.5, F12.5, F13.5, F14.5, F15.5, F16.5, F17.5, F18.5, and F19.5; ICD-9 codes 291 and 292). We used a hierarchical approach to psychosis in the analysis with those having a diagnosis of schizophrenia and related psychoses coded as “schizophrenia and related psychoses”; any diagnosis of affective psychoses with no diagnosis of schizophrenia and related psychoses was coded as “affective psychoses,” and “substance-related psychoses” coded in the absence of the other two groups. This hierarchical approach was applied on the same episode of care and also across the entire study period. From 86,461 individuals selected from the NSW’s APDC and EDDC from July 2001 to December 2012 as having psychosis we used a 5-year window from July 2001 to June 2006 in which no psychosis diagnosis was recorded in any of the two data collections (APDC and EDDC). We also used the MH-AMB data collection to determine whether any psychosis diagnosis record of the selected individuals existed before July 2006. Those from the first diagnosis cohort with any psychosis-related presentation in the MH-AMB data collection before July 2006 were removed from the first diagnosis cohort. Those with a psychosis-related diagnosis between July 2006 and December 2012 and before the diagnosis dates determined from the APDC or EDDC in the MH-AMB data collection, were considered as having a FDP for that individual (*n* = 38,489). This approach had previously been used in a study where the factors associated with a FDP in prison were determined (19).

#### Mental health service contact in the community

We considered any psychosis-related diagnosis in hospital (APDC) and emergency department (EDDC) and any contact in the community mental health service (MH-AMB data collection) following release from prison or discharge from hospital as a mental health service contact in the community. Mental health service contacts occurring in prison following a FDP were not considered in this study as the focus was on post-release engagement with mental health services.

#### Offending

Offences were defined as convictions, as recorded in the RoD and coded according to the Australian and New Zealand Standard Offence Classification (ANZSOC) (15). Offences that did not result in convictions were not included in this study.

### Data extraction

We extracted data on the type of psychosis, age at diagnosis, diagnosis episode start date and end date, gender, Aboriginality (yes, no, and unknown), marital status (married including *de facto*, single and missing or unknown), date of birth, and statistical local area (SLA) from the APDC and EDDC. Single marital status includes all individuals who were single, widowed, divorced, and permanently separated. The index of relative socioeconomic disadvantage (IRSD) is used to rank the socioeconomic status in each SLA by the Australian Bureau of Statistics (20). IRSD is one of the four socioeconomic indexes for areas (SEIFA) which can be used to rank the socioeconomic status in each geographic area, using data on income, education, employment, occupation, and housing. The lowest rank indicates the most disadvantaged area and the highest rank the most advantaged area. We categorised areas into disadvantaged (score 1–5) and advantaged (score 6–10).

State-wide information on mental health assessment, treatment, rehabilitation, or care of non-admitted patients in mental health day programs, psychiatric outpatient, and outreach services were extracted from the MH-AMB data collection. Information extracted from the RoD was on the principal offence type, offence date, and age at the time of the offence in the case of a court appearance. The start and end dates of prison episodes of the study population were collected from NSW OIMS data collection. The date of death was extracted from the NSW RBDM data collection.

### Statistical analysis

Socio-demographic characteristics of the study population were examined at the time of the FDP. We compared the characteristics of both the FDP groups using chi-square analyses. Kaplan–Meier survival curves were used to compare the time to contact mental health services following release or discharge by the study groups. *P*-values were presented for the log-rank tests. Cox regression models were used to examine the predictors for the mental health service contacts following release or discharge. We reported adjusted hazard ratios (aHRs) from the multivariable model along with the univariable analysis. We also used frequencies, median and interquartile range to describe the characteristics of mental health service contacts of the study population regarding their criminal history. In an additional sub-group analysis, we examined the length of stay in prison by post-release mental health service contacts in those with a FDP in prison. Data were analysed using SAS Version 9⋅4 and STATA 14.0 (College Station, TX, USA).

### Ethics approvals

Approvals were obtained from the NSW Population and Health Services Research Ethics Committee (HREC/15/CIPHS/17), Justice Health and Forensic Mental Health Network (G324/14), Corrective Services NSW (D15/138715), Cancer Institute NSW (2015/05/586), and the NSW Aboriginal Health and Medical Research Council (1089/15).

## Results

### Socio-demographic characteristics of the study population and their mental health service contact post release or discharge

Characteristics of the study population are shown in Table 1. A total of 25,402 individuals (54.1% men) were identified with a FDP in prison or hospital in NSW between July 2006 and December 2011 and were released or discharged from prison or hospital during this period. Of these, around 2% (*n* = 492) individuals had a FDP in prison and 24,910 in any hospital. FDP prevalence was higher in men in prison than women (3.1% vs. 0.6%). These individuals were followed up until December 2012 with a median follow-up of 25 days for the prison group and 26 days for the hospital group. In the overall analysis, a higher percentage of Aboriginal people were present in the prison group compared to the hospital group (28.9 vs. 5.8%, *p* < 0.001). Psychosis type was significantly different in the prison group compared to the hospital group (79.1, 5.5, 15.4% vs. 67.7, 19.6, 12.7% with *p* < 0.001 for individuals with schizophrenia and related psychoses, affective psychoses and substance-related psychoses respectively). A higher proportion of individuals in the younger and middle age group were in the prison group compared to those in the hospital group (20.5, 38.0, 26.9, 14.6% vs. 18.8, 20.4, 18.2, 42.6% with *p* < 0.001 in individuals with age <25, 25–34, 35–44, and 45+ years, respectively). Age groups were determined at the time of release from prison or discharge from hospital. Most of those with a FDP in prison were from disadvantaged postcodes compared to half of the individuals in the hospital group (87.0 vs. 50.3%, *p* < 0.001), and married marital status was higher in the hospital group compared to the prison group (25.4 vs. 16.7%, *p* < 0.001).

**TABLE 1:** Characteristics of individuals with a FDP^a^ in prison or hospital in NSW (*n* = 25,402), July 2006–December 2011.

Characteristics	Total (*n* = 25,402)			Men (*n* = 13,742; 54.1%)			Women (*n* = 11,660; 45.9%)		
					
	FDP in prison (*n* = 492; 1.9%)	FDP in hospital (*n* = 24,910; 98.1%)		FDP in prison (*n* = 424; 3.1%)	FDP in hospital (*n* = 13,318; 96.9%)		FDP in prison (*n* = 68; 0.6%)	FDP in hospital (*n* = 11,592; 99.4%)	
	
	*n* (%)	*n* (%)	*P*-value	*n* (%)	*n* (%)	*P*-value	*n* (%)	*n* (%)	*P*-value
**Aboriginal**			<0.001			<0.001			<0.001
No	350 (71.1)	23,462 (94.2)		312 (73.6)	12,475 (93.7)		38 (55.9)	10,987 (94.8)	
Yes	142 (28.9)	1,448 (5.8)		112 (26.4)	843 (6.3)		30 (44.1)	605 (5.2)	
**Psychosis type**			<0.001			<0.001			0.001
Schizophrenia and related psychoses	389 (79.1)	16,874 (67.7)		337 (79.5)	9,018 (67.7)		52 (76.5)	7,856 (67.8)	
Affective psychoses	27 (5.5)	4,867 (19.6)		22 (5.2)	2,093 (15.7)		5 (7.3)	2,774 (23.9)	
Substance-related psychoses	76 (15.4)	3,169 (12.7)		65 (15.3)	2,207 (16.6)		11 (16.2)	962 (8.3)	
**Age groups**			<0.001			<0.001			<0.001
<25	101 (20.5)	4,688 (18.8)		92 (21.7)	3,012 (22.6)		9 (13.2)	1,676 (14.5)	
25–34	187 (38.0)	5,077 (20.4)		159 (37.5)	3,055 (22.9)		28 (41.2)	2,022 (17.4)	
35–44	132 (26.9)	4,529 (18.2)		110 (25.9)	2,515 (18.9)		22 (32.4)	2,014 (17.4)	
45+	72 (14.6)	10,616 (42.6)		63 (14.9)	4,736 (35.6)		9 (13.2)	5,880 (50.7)	
**SEIFA**			<0.001			<0.001			<0.001
Advantaged	50 (10.1)	10,447 (41.9)		38 (8.9)	5,360 (40.2)		12 (17.6)	5,087 (43.9)	
Disadvantaged	428 (87.0)	12,518 (50.3)		373 (88.0)	6,707 (50.4)		55 (80.9)	5,811 (50.1)	
Missing or unknown	14 (2.9)	1,945 (7.8)		13 (3.1)	1,251 (9.4)		1 (1.5)	694 (6.0)	
**Marital status**			<0.001			<0.001			<0.001
Married (including *de facto*)	82 (16.7)	6,335 (25.4)		74 (17.4)	2,866 (21.5)		8 (11.7)	3,469 (29.9)	
Single	323 (65.6)	16,790 (67.4)		281 (66.3)	9,335 (70.1)		42 (61.8)	7,455 (64.3)	
Missing or unknown	87 (17.7)	1,785 (7.2)		69 (16.3)	1,117 (8.4)		18 (6.5)	668 (5.8)	
**Mental health service contact following release from prison or discharge from hospital**			0.651			0.532			0.521
Yes	353 (71.8)	18,101 (72.7)		301 (71.0)	9,638 (72.4)		52 (76.5)	8,463 (73.0)	
No	139 (28.2)	6,809 (27.3)		123 (29.0)	3,680 (27.6)		16 (23.5)	3,129 (27.0)	

^*a*^FDP, First diagnosis of psychosis.

More than two thirds from both groups had at least one mental health service contact following release from prison or discharge from hospital (71.8 vs. 28.2% in prison group and 72.7 vs. 27.3% in hospital group) with most contacts (80.2%) occurring with community-based mental health services, 19.0% with hospital contacts, and 0.8% with emergency room presentations between July 2006 and December 2012.

Overall, there were 54.1% of men in the study population with FDP prevalence higher in men in prison than women (3.1 vs. 0.6%). In the gender specific analysis with socio-demographic variables and mental health service contact, the same trend was observed in both men and women.

### Time to mental health service contacts following prison release or hospital discharge

**FIGURE 1:**
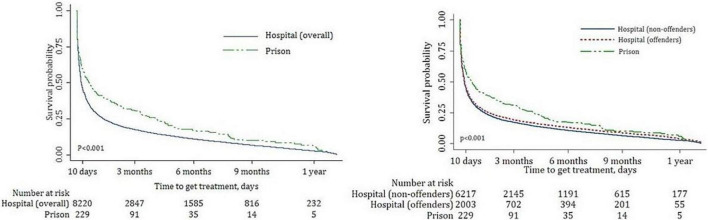
Time to contact mental health services following release from prison and discharge from hospital Kaplan–Meier survival curves prison vs. hospital (overall) group **(A)** and prison vs. hospital (offenders) vs. hospital (non-offenders) group **(B)**.

We further divided the hospital FDP group into those with and without previous offences (21.0% (*n* = 5,331) had a prior criminal conviction). In both the prison and hospital groups, around 90% of those who received at least one mental health service contact following release from prison (*n* = 353) or discharge from hospital (*n* = 18,101) had their first mental health service contact within 12 months of release or discharge. Kaplan–Meier curves (Figure 1) showed the hospital FDP group were significantly more likely to have contact with mental health services following discharge compared to the prison FDP group in the 12 months post-release or post-discharge period (log-rank test, *p* < 0.001). A similar pattern was observed in time to post discharge contact with mental health services in the hospital FDP group between those with and without prior convictions (Figure 1).

### Factors associated with *no mental* health service contact

Factors associated with *no* mental health service contact following release or discharge from prison or hospital are shown in Table 2. Results from both univariable and multivariable analysis adjusted by all variables are presented in the table. In both the univariable and multivariable analyses, individuals with a FDP in prison were more likely to have no contact with mental health services post-prison release compared to those in hospital (*HR* = 2.90, 95% CI: 2.45–3.44, *p* < 0.001 and *aHR* = 2.65, 95% CI: 2.22–3.15, *p* < 0.001). Similarly, those with a FDP in prison were three times more likely to have *no contact* with mental health services post-prison compared to those in hospital with no prior criminal conviction (*HR* = 3.14, 95% CI: 2.66–3.72, *p* < 0.001 and *aHR* = 3.05, 95% CI: 2.56–3.63, *p* < 0.001). Also, those with a FDP in hospital with a prior criminal conviction were more likely to have *no contact* with mental health services post-discharge compared to those in hospital with no prior criminal conviction (*HR* = 1.62, 95% CI: 1.52–1.72, *p* < 0.001 and *aHR* = 1.60, 95% CI: 1.49–1.71, *p* < 0.001).

**TABLE 2:** Overall and gender specific hazard ratios (univariable and multivariable) for no mental health service contact following release or discharge from prison or hospital (*n* = 25,402).

	Overall (*n* = 25,402)					Men (*n* = 13,742) (54.1%)					Women (*n* = 11,660) (45.9%)				
			
	*n* (%)	*HR* (95% CI)	*P*-value	*aHR* (95% CI)	*P*-value	*n* (%)	*HR* (95% CI)	*P*-value	*aHR* (95% CI)	*P*-value	*n* (%)	*HR* (95% CI)	*P*-value	*aHR* (95% CI)	*P*-value
**FDP groups**															
Hospital (overall)	24,910 (98.1)	1		1		13,318 (96.9)	1		1		11,592 (99.4)	1		1	
Prison	492 (1.9)	2.90 (2.45–3.44)	<0.001	2.65 (2.22–3.15)	<0.001	424 (3.1)	2.63 (2.19–3.15)	<0.001	2.54 (2.10–3.05)	<0.001	68 (0.6)	2.92 (1.78–4.78)	<0.001	2.61 (1.58–4.33)	<0.001
**FDP groups**															
Hospital (non-offenders)	19,579 (77.1)	1		1		9,257 (67.4)	1		1		10,322 (88.5)	1		1	
Hospital (offenders)	5,331 (21.0)	1.62 (1.52–1.72)	<0.001	1.60 (1.49–1.71)	<0.001	4,061 (29.6)	1.55 (1.44–1.67)	<0.001	1.58 (1.46–1.71)	<0.001	1,270 (10.9)	1.63 (1.43–1.85)	<0.001	1.63 (1.41–1.87)	<0.001
Prison	492 (1.9)	3.14 (2.66–3.72)	<0.001	3.05 (2.56–3.63)	<0.001	424 (3.0)	2.92 (2.44–3.51)	<0.001	2.93 (2.43–3.54)	<0.001	68 (0.6)	3.03 (1.85–4.96)	<0.001	2.94 (1.78–4.87)	<0.001
**Gender**															
Women	11,660 (45.9)	1		1		–	–		–		–	–		–	
Men	13,742 (54.1)	1.18 (1.13–1.24)	<0.001	1.11 (1.05–1.16)	<0.001	–	–	–	–	–	–	–	–	–	–
**Age at release or discharge**															
<25	4,789 (18.9)	1		1		3,104 (22.6)	1		1		1,685 (14.4)	1		1	
25–34	5,264 (20.7)	0.96 (0.88–1.04)	0.343	0.97 (0.89–1.06)	0.532	3,214 (23.4)	0.94 (0.85–1.05)	0.273	0.95 (0.86–1.06)	0.355	2,050 (17.6)	0.98 (0.85–1.13)	0.752	1.00 (0.86–1.15)	0.991
35–44	4,661 (18.4)	0.88 (0.80–0.96)	0.003	0.91 (0.83–0.99)	0.029	2,625 (19.1)	0.87 (0.78–0.97)	0.014	0.88 (0.78–0.98)	0.021	2,036 (17.5)	0.90 (0.77–1.04)	0.143	0.94 (0.81–1.09)	0.417
45+	10,688 (42.1)	1.04 (0.96–1.11)	0.354	1.22 (1.12–1.31)	<0.001	4,799 (34.9)	0.99 (0.90–1.09)	0.873	1.11 (1.00–1.23)	0.043	5,889 (50.5)	1.18 (1.04–1.33)	0.008	1.34 (1.18–1.52)	<0.001
**Aboriginal**															
No	23,812 (93.7)	1		1		12,787 (93.1)	1		1		11,025 (94.6)	1		1	
Yes	1,590 (6.3)	1.49 (1.34–1.65)	<0.001	1.25 (1.12–1.40)	<0.001	955 (6.9)	1.44 (1.26–1.65)	<0.001	1.19 (1.03–1.37)	0.016	635 (5.4)	1.50 (1.27–1.78)	<0.001	1.37 (1.14–1.63)	0.001
**Psychosis type**															
Schizophrenia and related Psychoses	17,263 (68.0)	1		1		9,355 (68.1)	1		1		7,908 (67.8)	1		1	
Affective psychoses	4,894 (19.3)	0.84 (0.79–0.89)	<0.001	0.86 (0.81–0.92)	<0.001	2,115 (15.4)	0.86 (0.78–0.94)	0.002	0.86 (0.78–0.95)	0.002	2,779 (23.8)	0.84 (0.77–0.92)	<0.001	0.87 (0.79–0.95)	0.002
Substance-related psychoses	3,245 (12.7)	1.19 (1.11–1.27)	<0.001	1.12 (1.04–1.20)	0.002	2,272 (16.5)	1.17 (1.08–1.26)	<0.001	1.09 (1.01–1.19)	0.038	973 (8.4)	1.15 (1.02–1.29)	0.020	1.17 (1.03–1.33)	0.013
**SIEFA**															
Advantaged	10,497 (41.3)	1		1		5,398 (39.3)	1		1		5,099 (43.7)	1		1	
Disadvantaged	14,905 (58.7)	1.06 (1.01–1.11)	0.025	1.01 (0.96–1.06)	0.625	8,344 (60.7)	1.10 (1.03–1.18)	0.003	1.05 (0.98–1.12)	0.133	6,561 (56.3)	0.98 (0.92–1.06)	0.648	0.96 (0.90–1.04)	0.317
**Marital status**															
Married	6,417 (25.3)	1		1		2,940 (21.4)	1		1		3,477 (29.8)	1		1	
Single	18,985 (74.7)	1.01 (0.96–1.07)	0.678	0.97 (0.91–1.02)	0.209	10,802 (78.6)	0.92 (0.86–0.99)	0.024	0.86 (0.80–0.93)	<0.001	8,183 (70.2)	1.09 (1.01–1.18)	0.026	1.07 (0.99–1.16)	0.075

Other factors associated with an increased likelihood of not having contact with mental health services post-release or post-discharge were: male gender (*HR* = 1.18, 95% CI: 1.13–1.24, *p* < 0.001 and *aHR* = 1.11, 95% CI: 1.05–1.16, *p* < 0.001), Aboriginal heritage (*HR* = 1.49, 95% CI: 1.34–1.65, *p* < 0.001 and *aHR* = 1.25, 95% CI: 1.12–1.40, *p* < 0.001), and having a diagnosis of substance-related psychoses compared to schizophrenia and related psychoses (*HR* = 1.19, 95% CI: 1.11–1.27, *p* < 0.001 and *aHR* = 1.12, 95% CI: 1.04–1.20, *p* = 0.002).

In the multivariable analysis, older age (over 44 years) was associated with an increased likelihood of *not having contact* with mental health services compared to the younger group (<25 years of age) (*aHR* = 1.22, 95% CI: 1.12–1.31, *p* < 0.001) whereas it was non-significant in the univariable analysis. Missing or unknown SEIFA (7.7%) and missing or unknown marital status (12.5%) was further adjusted for in the multivariable analysis. In the univariable analysis individuals from disadvantaged postcodes were more likely to have no contact with mental health services compared to those from advantaged postcodes (*HR* = 1.06, 95% CI: 1.01–1.11, *p* = 0.025). However, the risk increased was not statistically significant in the multivariable analysis. Marital status was not associated with post release or discharge contact with mental health services in the overall analysis.

In the gender specific analysis, a similar trend was found in both men and women in both univariable and multivariable analysis in terms of setting of the FDP, gender, aboriginality and psychosis type. In Contrast with the overall analysis, older women with a FDP were more likely to have no contact with mental health services compared to the younger group (*HR* = 1.18, 95% CI: 1.04–1.33, *p* = 0.008) in the univariable analysis. Disadvantaged postcode was not associated with not having a mental health contact in women. Unlike the overall analysis, women with single marital status were more likely to have no contact with mental health services (*HR* = 1.09, 95% CI: 1.01–1.18, *p* = 0.026) compared to those women who were married in the univariable analysis whereas it was the opposite for men in both the univariable and multivariable analysis (*HR* = 0.92, 95% CI: 0.86–0.99, *p* = 0.024 and *aHR* = 0.86, 95% CI: 0.80–0.93, *p* < 0.001).

### Prior offence and mental health service contacts

Predictably, the median length of stay in prison after a FDP was longer at 3 months (IQR = 23 days–7 months) compared with a median length of stay in a hospital of 5 days (IQR = 1–17 days) for those with a prior criminal conviction and 10 days (IQR = 3–23 days) for those who had no previous criminal offence record (Table 3). 46.2, 55.4, and 47.5% of individuals released from prison, discharged from hospital with and without a prior criminal conviction, had contact with a *community-based* mental health service within 3 months of release or discharge from prison or hospital. Among those who had mental health service contact, over 60% from all three groups received more than 10 mental health service contacts in the follow-up period (65.4, 68.6, and 61.3%, respectively).

**TABLE 3:** Characteristics of mental health service contacts of the study population by prior offence or prison episodes in NSW, July 2006–December 2012 (*n* = 25,402).

Characteristics	FDP^a^ in prison (*n* = 492) (1.9%)	FDP in hospital (offenders) (*n* = 5,331) (21.0%)	FDP in hospital (non-offenders) (*n* = 19,579) (77.1%)	*P*-value
			
	*n* (%)	*n* (%)	*n* (%)	
Median length of stay in prison or hospital (IQR) following the FDP	3 months (23 days–7 months)	5 days (1–17 days)	10 days (3–23 days)	<0.001
**Mental health service contact following discharge or release (July 2006–December 2012)**				<0.001
No mental health service contact	139 (28.2)	1,196 (22.4)	5,613 (28.7)	
Hospital and emergency only	67 (13.6)	700 (13.1)	2,892 (14.8)	
Community-based mental health service contact-brief (≤3 months)	227 (46.2)	2,950 (55.4)	9,273 (47.5)	
Community-based mental health service contact—extended (>3 months)	59 (12.0)	485 (9.1)	1,801 (9.2)	
Median time to first mental health service contact (IQR) following discharge or release	9 days (1 day–61 days)	7 days (2–37 days)	9 days (2–55 days)	<0.001
**No. of mental health service contact following release or discharge (for those who got any mental health service)**				<0.001
1 contact	46 (13.0%)	250 (6.1%)	1,321 (9.5%)	
2–10 contacts	76 (21.5%)	1,047 (25.3%)	4,079 (29.2%)	
>10 contacts	231 (65.4%)	2,838 (68.6%)	8,566 (61.3%)	
Median no. of mental health service contacts following release or discharge (IQR)	25 (5–88)	24 (7–70)	18 (5–53)	<0.001

^a^FDP, First diagnosis of psychosis.

### Mental health service contacts post-prison

**FIGURE 2:**
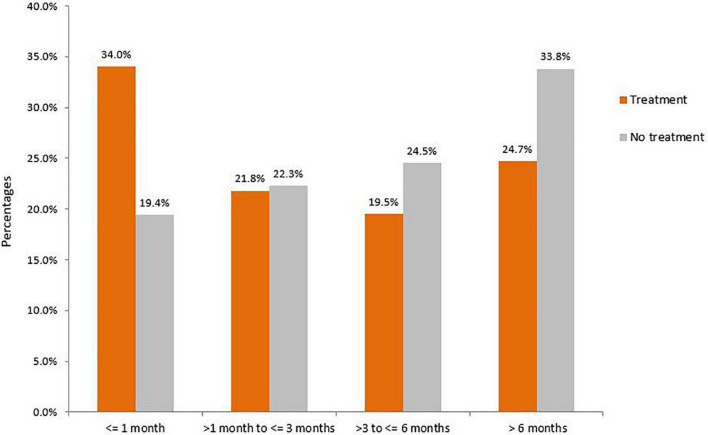
Duration of incarceration and post-release engagement by mental health services (*n* = 492).

In a sub-group analysis of the prison FDP group (*n* = 492), mental health service contact following release was associated with length of stay in prison following the FDP (Figure 2). Those who stayed in prison for the shortest post FDP (1 month or less) were more likely to have contact with mental health services post-release (34.0%) than those with longer stays in prison (21.8, 19.5, and 24.7% for those who stays 1–3 months, 3–6 months, and more than 6 months, respectively).

## Discussion

Our findings show that most of those with a FDP either in prison or hospital had at least one contact with mental health services following release from prison or discharge from hospital. These contacts were either as an admitted patient (19.0%) or emergency room presentation (0.8%) or with community mental health services (80.2%). However, less than 60% of those exiting prison or hospital had contact with *community-based* mental health services within 3 months of being released or discharged. These findings suggest that individuals with psychosis are often lost to mental health service follow-up in the community following their first diagnosis regardless of the setting, and so do not receive short-term support from community-based mental health services. Further, those with a FDP in prison were significantly less likely to be in contact with any mental health services compared to those with a FDP in hospital upon release, regardless of their offending history. However, in the multivariable analysis, the results were adjusted by all the variables including gender, age, Aboriginality, and psychosis type which were also associated with less contact in the community. The above findings support the need to assertively follow-up those with serious mental illnesses such as psychosis exiting prisons.

The transition period from prison to the community is a critical time for this population to successfully integrate back into society and mental health contact appears to have a key role to play in the rehabilitative process and reduction of recidivism (21). Previous studies have reported the challenges in mentally ill individuals with prior prison episodes and psychiatric in-patients successfully re-entering the community following release or discharge (13, 22). According to our study, the longer the incarceration episode following diagnosis, the less likely individuals were to have contact with mental health services which likely reflects the dislocation that incarceration imposes on those in prisons and may be amplified in those with more serious mental illnesses resulting in reduced access to community mental health services. It also suggests that prison mental health services, community mental health services, and social/welfare support services need to synergise to ensure that those released from prison have a soft landing when transitioning from prison back to the community, particularly in regard to receiving post-prison treatment. This is likely to be challenging as it is not always clear where a person released from prison will be living when they return to the community. To this end, probation and parole services could potentially play a significant role in facilitating and brokering the transition from prison to the community and ensuring that mental health treatment is received. Specialist and dedicated workers may also be needed with forensic experience to help navigate the route back to the community for this vulnerable population group. At a minimum, both community mental health service staff and those responsible for post-release supervision should be trained to manage and support this group. In NSW approximately, 80% of those leaving prisons do so with some sort of community supervision requirement which ensures they are, at a minimum, in contact and visible to the community justice system (Caruana R, Assistant Commissioner, Community Corrections, Corrective Services NSW, personal communication). However, given that most community corrections and probation officers who manage ex-prisoners released on licence have backgrounds in social work, they are likely to be at a disadvantage in terms of effectively navigating the mental health system. Possible solutions to overcome this could be better training of this group in terms of mental health treatment options, embedding mental health nurses with experience of community mental health services within community corrections offices, or the establishment of a separate service to manage those exiting prisons with serious mental health conditions.

According to our study, individuals with a FDP in prison and in hospital with a prior offence history were less likely to have mental health service contacts following release or discharge compared to those individuals diagnosed in hospitals with no prior offence. This could be related to factors such as a reluctance on the part of community mental health services to engage with those who have criminal histories, a tendency for the offender population to avoid contact, or challenging social circumstances post-release from prison. This finding is similar to a study that compared several pathways to mental health service use in the community among ex-prisoners in Queensland, Australia which found fewer contacts with mental health services in the community (8). We also found that those with prior offence records tended to have shorter stays in hospital compared to those with no prior offence which might be attributable to factors such as bias on the part of mental health services, and/or difficulty posed to mental health services in retaining this population in hospital, but this needs further investigation. A diagnosis of substance induced psychosis could also explain their limited engagement with mental health services- the effects of recurrent substance intoxication on motivation to engage with services, or reluctance by services to manage these population group as substance induced psychosis is not necessarily a chronic enduring psychotic disorder that requires ongoing treatment.

This study found that among those with mental health service contact, those who were diagnosed in prison and in a hospital with a prior offence record received a greater number of mental health service contacts compared to those diagnosed in hospital with no prior offence. This finding may indicate that ex-prisoners or offenders have more severe mental health problems requiring increased contact with services in the community.

Gender, Indigenous status, and older age and a single marital status in women were also identified as barriers in terms of receiving mental health service contacts among newly diagnosed individuals regardless of their place of diagnosis and offending behaviour. Men were less likely to have contact with mental health services compared to women. Several studies have similarly found that men are less likely than women to seek professional or community mental health treatment (23, 24) and given the higher rate of offending amongst males as a group, this strengthens the argument for better integration into the community for this group. According to our study, individuals of Aboriginal heritage with a FDP regardless of the setting were less likely to have mental health service contact compared to the non-Aboriginal people. This adds to the evidence for culturally tailored mental health treatment programs for those of Aboriginal heritage and cultural safety within mental health services (25). Special attention should be paid to older people with a FDP especially women as they may face additional difficulties in receiving mental health services compared to the younger people. We found that individuals with drug-related psychosis were less likely to seek support post release or discharge from hospital which could reflect a reluctance by mental health services to accommodate these individuals in the community.

Studies support the notion that effective discharge planning is important in reducing re-hospitalisation and involvement in post-discharge care among mentally ill people is essential (26). Along with the implementation of an appropriate release or discharge plan, effective interventions should include education for both patients and caregivers, and appropriate management of these individuals by justice and health staff involved in transition planning.

## Limitations

This study did not include those diagnosed in private clinics or treated by general practitioners. It may have resulted in some individuals being missed. However, advice from psychiatrists is that most individuals do intersect with the public system at some point and thus this number is likely to be minimal. Data were only available from 2001 to 2012 hence we could not examine diagnostic information prior to 2001. Further, individuals having a diagnosis of psychosis outside the state or country would have been missed in this state-based linkage study covering NSW only. Offending data used in this study covered convictions only thus self-reported offences might have gone unreported.

## Conclusion

Continuation of mental health service contact for those with a FDP following release from prison or discharge from hospital is likely to contribute to better reintegration into the community and improved health and justice outcomes. Community support and social acceptance through financial, social, and mental stability is the key to keeping these individuals away from revolving around the criminal justice and hospital system and improving the quality of life for ex-prisoners.

## Data availability statement

There is an adequate plan to protect the confidentiality of data. The data has been stored on a secure server located at the School of Population Health, University of New South Wales which is in a password protected de-identified format that is only accessible to members of the research team. Requests to access these datasets should be directed to TB, tbutler@unsw.edu.au.

## Ethics statement

The studies involving human participants were reviewed and approved by NSW Population and Health Services Research Ethics Committee (HREC/15/CIPHS/17), Justice Health and Forensic Mental Health Network (G324/14), Corrective Services NSW (D15/138715), Cancer Institute NSW (2015/05/586), and NSW Aboriginal Health and Medical Research Council (1089/15). Written informed consent from the participants’ legal guardian/next of kin was not required to participate in this study in accordance with the national legislation and the institutional requirements.

## Author contributions

TB, NC, OA, HW, AK, and AA planned and designed this study. NC analysed and wrote the manuscript. All authors contributed to the article and agreed to submit this version of the manuscript.
